# Management of fixed flexion contracture in primary total knee arthroplasty: recent systematic review

**DOI:** 10.1051/sicotj/2024007

**Published:** 2024-03-26

**Authors:** Elliot Sappey-Marinier, Andréa Fernandez, Jobe Shatrov, Cécile Batailler, Elvire Servien, Denis Huten, Sébastien Lustig

**Affiliations:** 1 Département de chirurgie orthopédique et de médecine du sport, FIFA medical center of excellence, Hôpital de la Croix-Rousse, Centre Hospitalier Universitaire de Lyon Lyon France; 2 Univ Lyon, Université Claude Bernard Lyon 1, IFSTTAR, LBMC UMR_T9406 Lyon France; 3 Service de chirurgie Orthopédique, Centre chirurgical Emile Gallé, Centre Hospitalier Universitaire de Nancy Nancy France; 4 LIBM – EA 7424, Interuniversity Laboratory of Biology of Mobility, Université Claude Bernard Lyon 1 Lyon France; 5 Chirurgie Orthopédique, Réparatrice et Traumatologique, Centre Hospitalier Universitaire de Rennes Rennes France

**Keywords:** Total knee arthroplasty, Fixed flexion contracture, Posterior capsule release, Postoperative rehabilitation, Distal femoral cuts

## Abstract

*Introduction*: This study aimed to systematically review the literature and identify the surgical management strategy for fixed flexion contracture in primary total knee arthroplasty (TKA) surgery, pre-, intra-, and post-operatively. Secondary endpoints were etiologies and factors favoring flexion contracture. *Materials and methods*: Searches were carried out in November 2023 in several databases (Pubmed, Scopus, Cochrane, and Google Scholar) using the following keywords: “flexion contracture AND TKA”, “fixed flexion deformity AND TKA”, “posterior capsular release AND TKA”, “posterior capsulotomy in TKA”, “distal femoral resection AND TKA”. Study quality was assessed using the STROBE checklist and the Downs and Black score. Data concerning factors or strategies leading to the development or prevention of flexion contracture after TKA were extracted from the text, figures, and tables of the included references. The effect of each predictive factor on flexion contracture after TKA was recorded. *Results*: Thirty-one studies were identified to meet the inclusion and exclusion criteria. These studies described a variety of preoperative and intraoperative factors that contribute to the development or correction of postoperative flexion contracture. The only clearly identified predictor of postoperative flexion contracture was preoperative flexion contracture. Intraoperative steps described to correct flexion contracture were: soft-tissue balancing (in posterior and medial compartments), distal femoral resection, flexion of the femoral component, and posterior condylar resection. However, no study has investigated these factors in a global model. *Discussion*: This review identified various pre-, intra-, and post-operative factors predictive of post-operative flexion contracture. In practice, these factors are likely to interact, and it is therefore crucial to further investigate them in a comprehensive model to develop an algorithm for the management of flexion contracture.

Level of evidence: IV

## Introduction

Flexion contracture is common in knee arthropathies at the total knee arthroplasty (TKA) stage, accounting for up to 61% of cases for Tew and Forster [1]. Like varus or valgus, it is not a joint deformity but a stiffness, with a defect in extension sometimes associated with a defect in flexion. Frontal misalignments have been more extensively studied than flexion, the deleterious consequences of which are less well-known and the treatment less well codified. Beyond 5°, flexion contracture penalizes the functional score of a TKA [2] and above 15°, it is considered a significant cause of disability.

In the case of flexion contracture, the patient must contract the quadriceps to prevent the knee from slipping out of flexion during weight-bearing, which requires the quadriceps to work harder, leading to the anterior thigh and even knee pain. Indeed, Perry et al. [3] showed that quadriceps workload increased by 22–51% when flexion contracture was increased from 15° to 30°. Murphy et al. [4] have shown that energy expenditure is significantly increased from 20° of flexion contracture in patients with TKA versus 15° in subjects in a control group without TKA. In the case of flexion contracture, walking is done with the foot still flat on the ground, without rolling the step from heel to toe [5].

It must be managed during the operation and postoperatively with appropriate rehabilitation. The aim is to free up two quadrangular spaces of identical height for extension and flexion. Flexion contracture is a problem of the extension space, and the difficulty is to increase the insufficient height of the extension space to equalize it with that of the flexion space. Several empirical surgical algorithms have been proposed, including osteophyte removal, ligament release, and additional distal femoral resection, all of which alter the height of the joint line (JL) and expose it to frontal laxity in mid-flexion [6–9].

The prevalence of flexion contracture after TKA is estimated at 1.4–17% [10]. Many authors agree that flexion contracture, especially of 15° or more, significantly reduces walking ability and functional performance, and penalizes scores [5, 11, 12]. In addition, the ideal sagittal alignment is 0–5° immediately after surgery, as it becomes penalizing beyond 5° [13, 14].

There is only one systematic review on the topic, which attempts to propose a precise management of flexion contracture in TKA [15]. Thus, a recent systematic review of the literature seems necessary. The primary endpoint is the surgical management strategy for flexion contracture in primary TKA, pre-, intra- and post-operatively. Secondary endpoints are etiologies and factors favoring flexion contracture.

## Material and methods

### Literature search strategy

In this study, the Preferred Reporting Items for Systematic Reviews and Meta-Analyses (PRISMA) guidelines were followed [16]. Searches were carried out in November 2023 in several databases (Pubmed, Scopus, Cochrane, and Google Scholar) using the following keywords: “flexion contracture AND TKA”, “fixed flexion deformity AND TKA”, “posterior capsular release AND TKA”, “posterior capsulotomy in TKA”, “distal femoral resection AND TKA”. In addition, the references of all selected articles were checked to include all relevant articles. Two authors independently identified the articles according to the selection criteria described below. The approval of the ethics committee is not required in our institution for this type of study. No funding was received for this project.

### Selection criteria

The inclusion criteria for this recent systematic review included English-language articles published after 1990 that met the primary and secondary judgment criteria.

Exclusion criteria included articles on TKA revisions and partial prostheses. Studies on rheumatoid arthritis or hemophilic arthropathy were excluded. Clinical cases, abstracts, editorial letters, and systematic reviews were also excluded.

### Data extraction

Data concerning factors or strategies leading to the development or prevention of flexion contracture after TKA were extracted from the text, figures, and tables of the included references. The effect of each predictive factor on flexion contracture after TKA was recorded.

### Quality assessment of articles

Strengthening the Reporting of Observational Studies in Epidemiology (STROBE) was used to assess the structure, quality, and reporting of studies [17]. The study design was classified according to the NHMRC level of evidence classification. Studies were then assessed using the Downs and Black score [18].

### Statistical analysis

The included studies were divided into three main categories according to the factors investigated: preoperative factors, surgical steps, and surgical algorithms. Preoperative factors were variables associated with patients developing postoperative flexion contracture. Surgical steps were individual measurements employed intraoperatively to correct flexion contracture and prevent its incidence postoperatively. Studies of surgical algorithms assessed the success of sequences involving specific intraoperative steps employed to correct and prevent postoperative flexion contracture.

## Results

### Search results

**Figure 1:**
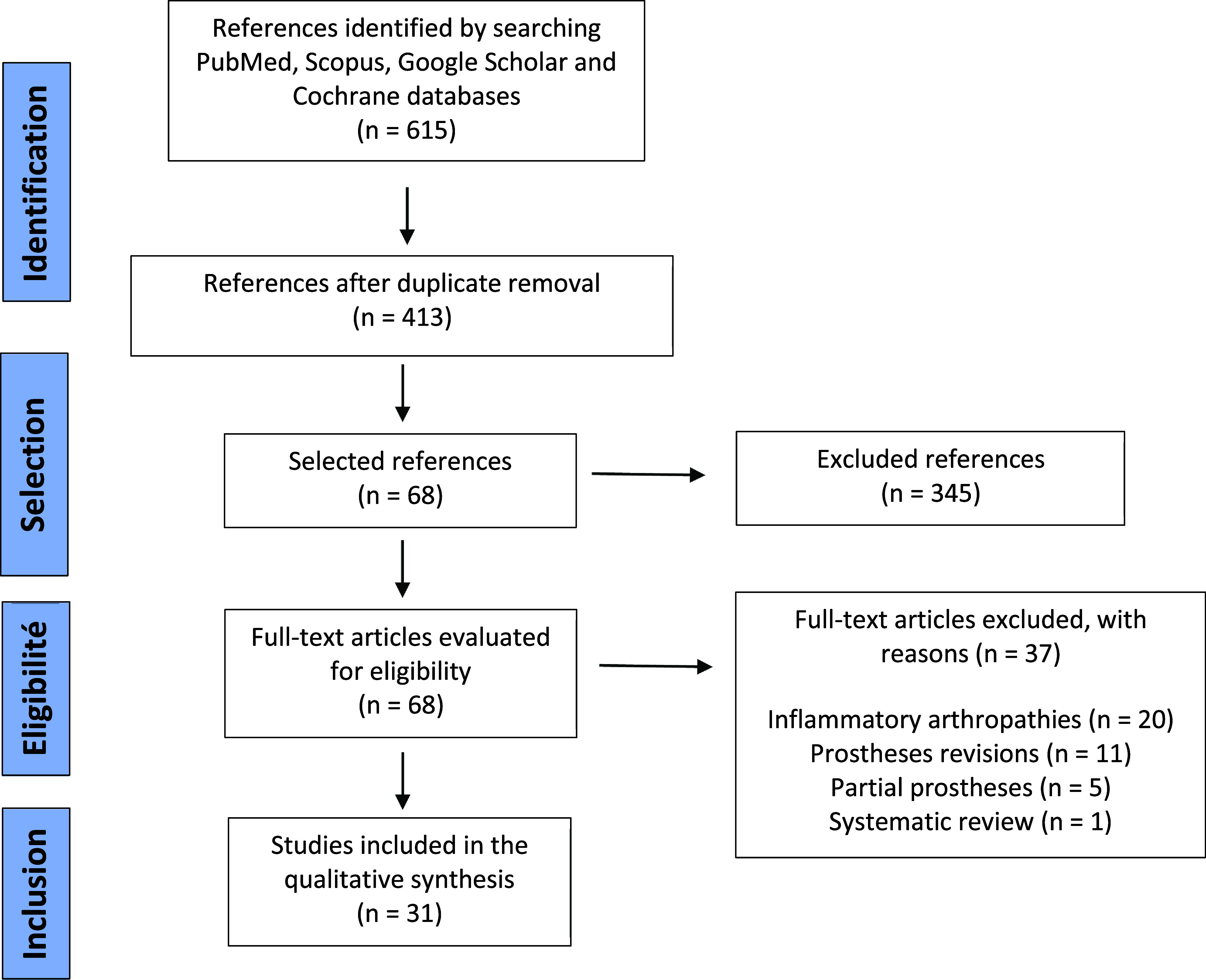
Flowchart.

The initial search identified 615 articles. Sorting of titles and abstracts resulted in 68 articles being retained for full-text review. After applying inclusion and exclusion criteria and searching the reference lists of included articles, 31 studies were selected for qualitative analysis (Figure 1).

The studies included varied in terms of design, techniques for measuring the extension range of motion, and follow-up (Table 1). One randomized controlled trial, 24 cohort studies, four case-control studies, and two studies describing a surgical technique were included. Knee mobility was assessed at various time points, from immediate post-operative to 120 months. Goniometers were used in 18 studies: four studies used computer navigation, two used radiographs, one study used intraoperative photographs and skin landmarks, and six did not indicate their method of assessment.

**Table 1:** Studies characteristics.

Authors	Year	Study design	Flexion contracture measurement technique	Postoperative flexion contracture measurement follow up	Downs and Black score
Kinoshita et al. [19]	2021	Cohort	Goniometer	6 month	17
Chai et al. [22]	2021	Cohort	NA	27 month	18
Matziolis et al. [23]	2020	Cohort	Navigation	Peri-operative	17
Leie et al. [24]	2019	Cohort	Navigation	Peri-operative	20
Okamoto et al. [25]	2019	Case-controlled	Goniometer	24 month	21
Kim et al. [26]	2017	Cohort	Navigation	Peri-operative	18
Okamoto et al. [27]	2016	Cohort	Goniometer	Peri-operative	16
Liu et al. [28]	2016	Cohort	Navigation	Peri-operative	21
Nagai et al. [29]	2015	Cohort	Radiographs	4 week	19
Okamoto et al. [30]	2014	Cohort	Radiographs	12 month	20
Bin Abd Razak et al. [31]	2014	Cohort	Goniometer	24 month	17
Debette et al. [32]	2014	Cohort	Goniometer	12 month	21
Koh et al. [20]	2013	Case-controlled	Goniometer	35 month (24–72 month)	18
Onodera et al. [33]	2013	Cohort	NA	NA	16
Meftah et al. [34]	2012	Cohort	NA	37 month (20–59 month)	16
Lustig et al. [21]	2012	Case-controlled	Goniometer	12 month	18
Su [12]	2012	Surgical technique	NA	NA	NA
Goudie et al. [11]	2011	Case-controlled	Goniometer	24 month	17
Smith et al. [35]	2010	Cohort	Photographs with skin markings	Peri-operative	20
McAllister and Stepanian [36]	2008	Cohort	Goniometer	12 month	20
Chaudhary et al. [37]	2008	RCT	Goniometer	24 month	22
Asano et al. [38]	2008	Cohort	Goniometer	12 month	14
Ritter et al. [13]	2007	Cohort	Goniometer	Minimum 72 month	17
Scuderi and Kochhar [39]	2007	Surgical technique	NA	NA	NA
Bellemans et al. [40]	2006	Cohort	Goniometer	24 month	18
Berend et al. [41]	2006	Cohort	Goniometer	38 month (1.6–77.0 month)	17
Bengs and Scott [42]	2006	Cohort	Goniometer	Peri-operative	21
Gatha et al. [43]	2004	Cohort	Goniometer	51 month (24–72 month)	16
Mihalko and Whiteside [44]	2003	Cohort	NA	70 month (12–120 month)	16
Whiteside and Mihalko [45]	2002	Cohort	Goniometer	24 month	17
Firestone et al. [46]	1992	Cohort	Goniometer	53 month	15

NA: Non available; RCT: randomized controlled trial.

### Studies quality

The quality of the included studies was determined by their NHMRC level of evidence. Only one study was level II, while 16 studies were level III and 17 were level IV. The median Downs and Black score was 18 out of a possible 32 points (Min–Max: 14–22).

### Studies result

#### Preoperative factors

Five of the included studies [11–13, 19–46] correlated patient preoperative factors with the incidence of postoperative flexion contracture. Predictors of postoperative flexion contracture were male gender, older age, and preoperative flexion contracture. The role of gender and age in the development of postoperative flexion contracture remains unclear. Ritter et al. [13] identified individual factors favoring post-operative flexion contracture of more than 10°: pre-operative flexion contracture of more than 5°, male gender (risk 2.3 higher than in women), age (risk increased by 35% per decade). A higher body mass index (BMI), on the other hand, reduced the risk (35% reduction in risk per 5 BMI units). Goudie et al. [11] found the same risk factors: male gender (2.6 times greater risk than females), pre-operative flexion contracture (2.3 times greater risk), and older age. However, BMI was not a risk factor. Kinoshita et al. [19] found a significantly greater risk of postoperative flexion in male patients with preoperative flexion. In contrast, a later study by Lustig et al. [21] disagreed with all factors, finding no significant difference between patients with and without flexion contracture of at least 5° at one year postoperatively in terms of gender or age. Similarly, Koh et al. [20] found preoperative flexion contracture to be the main factor in postoperative flexion contracture.

#### Surgical steps

For patients with flexion contracture, the included studies also examined the effect of surgical steps on flexion contracture. In particular, the role of distal femoral resection, femoral component flexion, and ligament releases were investigated.

##### Distal femoral resection

Increased bone resection of the distal femur has been studied by five separate groups [23, 26, 28, 35, 42], showing that greater resection of the distal femur restores a greater degree of extension.

Bengs and Scott [42], Smith et al. [35], Liu et al. [28], and Matziolis et al. [23] have used different approaches to evaluate the effect of distal femoral resection on extension. Using inserts of increasing thickness to mimic the effect of “excess” distal femur on the development of flexion contracture intraoperatively, Smith et al. [35] found that for every 2 mm of bone resected, approximately 3.6° of the extension was restored, while Liu et al. [28] found that with the first 2 mm of distal femur resection, 3.36° was restored, while Matziolis et al. [23] found a linear relationship of 2.2° ± 0.3° of extension restored per mm of femoral resection performed. At the same time, Bengs and Scott [42] found that for every 2 mm of bone resected, 9° of passive extension could be restored. Finally, Kim et al. [26] observed a restoration of 4.8° ± 0.1° of extension for every 2 mm of additional resection in the distal femur.

Studies performed with navigation [23, 26, 28] are more accurate. Overall, a gain of around 2° of extension per mm of additional resection of the distal femur can be assumed.

##### Femoral component flexion

Only two studies were found that examined the role of femoral component flexion on the development of postoperative flexion contracture. In the study by Lustig et al. [21], flexion greater than 3.5° was associated with a 2.9-fold increased risk of moderate flexion contracture. In the study by Okamoto et al. [25], femoral flexion was 7.3° ± 1.4° in cases with flexion contracture of 10° or more, compared with 4.2° ± 1.2° in cases with flexion contracture of less than 10°.

##### Soft tissue releases

Soft-tissue release appears to be one of the essential steps in the management of flexion contracture in TKA. This includes osteophyte removal, ligament release in the frontal plane, potential sacrifice of the posterior cruciate ligament (PCL), and release of the posterior capsule.

Kim et al. [26] used navigation to determine the influence of soft-tissue releases. Improvement was measured after each step: medial release reduced flexion contracture by 5.2° ± 2.8°, PCL sacrifice by a further 2.5° ± 2.2° and standard bone resections by 3.1° ± 3.2°. After the removal of the posterior osteophytes and with the trial parts in place, it was 2.7° ± 1.9°. Leie et al. [24] showed that the removal of osteophytes above the posterior femoral condyles increased extension by 2.7°–4.5°, depending on the size of the osteophytes.

Okamoto et al. [27] have shown that releasing the posterior capsule around the intercondylar notch can effectively widen the extension space and correct flexion contracture. Chai et al. [22] also demonstrated preoperative correction of flexion contracture by fusiform capsulectomy of the posterior capsule. However, these two studies do not indicate the degrees corrected by this posterior release.

### Surgical algorithms

Eleven studies [12, 22, 26, 32, 34, 39–41, 44–46] reported on the efficacy of intraoperative algorithms for flexion contracture correction. Although each study reported a slightly different surgical algorithm (see Table 2), the algorithms mainly consisted of osteophyte removal, notably posterior, followed by soft-tissue releases, sometimes including the posterior capsule, and, in the event of residual flexion contracture, additional recutting of the distal femur. These steps enabled the preoperative flexion contracture to be corrected to less than 5°.

**Table 2:** Description of studies using algorithms to correct flexion contracture, and the steps taken to correct this deformity.

Authors and year of publication	Different steps in the surgical algorithm
Chai et al. 2021 [22]	1. Frontal ligament balancing after osteophyte removal;
	2. Additional resection of distal femur up to 4 mm;
	3. Fusiform capsulectomy of posterior capsule.
Kim et al. 2017 [26]	1. Removal of osteophytes, release of the deep layer of the MCL and minimal release of the semimembranosus at its tibial insertion;
	2. Sacrifice of the PCL;
	3. Removal of posterior osteophytes and release of posterior capsule;
	4. Additional 2 mm distal femur resection.
Debette et al. 2014 [32]	1. Removal of osteophytes, release of the deep layer of the MCL and minimal release of the semimembranosus at its tibial insertion (varus knees) or release of the anterolateral capsule (valgus knees);
	2. Sacrifice of the PCL;
	3. Removal of posterior osteophytes and release of posterior capsule;
	4. Additional 2 mm distal femur resection;
	5. Additional tibial resection.
Meftah et al. 2012 [34]	1. Release the posteromedial capsule and PCL resection;
	2. Introduce a spacer to assess the superficial MCL, release the MCL using the pie crust technique;
	3. Manipulate with repeated valgus pressure while the spacer is in place, until 2–3 mm of “elastic flexibility” is achieved, with greater accepted laxity medially.
Su 2012 [12]	1. Frontal ligament balancing after osteophyte removal;
	2. Sacrifice of PCL;
	3 Additional resection of distal femur up to 6 mm; 4. Removal of posterior osteophytes and release of posterior capsule.
Scuderi and Kochhar 2007 [39]	1. Frontal ligament balancing after osteophyte removal;
	2. Sacrifice PCL (if flexion contracture >10°);
	3. Removal of posterior osteophytes and release of the posterior capsule;
	4. Additional resection of distal femur from 3 mm to 10 mm.
Bellemans et al. 2006 [40]	1. Frontal ligament balancing after osteophyte removal and additional 2 mm resection of the distal femur;
	2. Release of posterior capsule and gastrocnemius muscles;
	3. Additional distal femur resection up to 4 mm;
	4. Hamstring tenotomy.
Berend et al. 2006 [41]	1. Removal of osteophytes;
	2. Resection of distal femur;
	3. Release of PCL;
	4. Additional distal femur resection up to 4 mm, soft-tissue release.
Mihalko and Whiteside 2003 [44]	1. Release of posterior capsule;
	2. Additional resection of distal femur.
Whiteside and Mihalko 2002 [45]	1. Osteophyte removal and ligament balancing;
	2. Release of medial capsule in varus knees, followed by release of posterolateral capsule if necessary;
	3. Additional resection of the distal femur.
Firestone et al. 1992 [46]	1. Removal of osteophytes and foreign bodies;
	2. Additional resection of distal femur;
	3. Release of soft tissue in extension and flexion (posteromedial in varus knees, posterolateral in valgus knees).

MCL: medial collateral ligament; PCL: posterior cruciate ligament.

## Discussion

This systematic review identified potential surgical steps and decision-making algorithms for the management of flexion contracture in TKA. The main factor predisposing to flexion contracture after TKA is preoperative flexion contracture.

In the systematic review by An et al. [15], preoperative flexion contracture was the only favorable factor. Moreover, the greater the preoperative flexion contracture, the greater the risk of postoperative flexion contracture [47–49]. On the other hand, there is no consensus regarding gender, age, and BMI.

Soft tissue release is an essential step in flexion contracture management. This systematic review has identified the various steps involved, including the release of the posterior capsule and the removal of posterior osteophytes [24, 27, 50]. The capsule must be freed from the femur by 1–2 cm, which will lengthen it without destabilizing the knee [51]. If the space in extension is still insufficiently high, which may be the case in flexures of more than 25°, posterior release can be further increased by detaching the gastrocnemius tendon insertions flush with the bone. Finally, a capsulotomy can be performed, of which several techniques have been described [22, 52, 53]. In all cases, care must be taken with the vascular-nervous pedicle, whose relationship with the skeleton in extension and 90° flexion has been the subject of numerous studies [54, 55].

The PCL tightens in flexion and is therefore not normally retracted in flexion contracture unless significant posterior bone wear brings its two insertions together. In other cases, the PCL may be retained, even in flexion of more than 20° [41, 44, 45]. Kayani et al. [56] have recently demonstrated, in a series of robotic-assisted TKAs for osteoarthritis, that the PCL contributes to flexion contracture, and that sacrificing it helps to reduce some of the flexion contracture, by an average of 2.9° ± 1.6°, the more so the greater the preoperative flexion contracture (mean flexion was 6.3°). The greater the degree of flexion contracture, the greater the benefit of sacrificing the PCL. However, sacrificing the PCL increases the height of the flexion space more than the extension space in the medial compartment: by an average of 2.4 mm ± 1.5 versus an average of 1.3 mm ± 1.0, and even more so in the lateral compartment, by an average of 3.3 mm ± 1.6 versus an average of 1.2 mm ± 0.9, resulting in mediolateral laxity in flexion but not in extension.

It has been shown that sagittal positioning can influence the development of flexion contracture postoperatively [21, 25]. However, Antony et al. [57] found no correlation between flexion of the femoral component and postoperative flexion contracture.

An additional distal femoral resection has the advantage of greater simplicity. If the PCL is preserved, the resection should not exceed 2 mm, otherwise, the isometry of the preserved ligament will be altered too much [41, 45]. It is difficult to give a precise limit for posterior-stabilized TKAs (up to 10 mm [39] for a posterior-stabilized prosthesis). Some authors suggest over-resecting the distal femur by 2 mm as soon as the flexion contracture exceeds 10° [12, 40]. In this systematic review, it was shown that a gain of around 2° of the extension was obtained per mm of additional distal femur resection. Unfortunately, these additional resections increase frontal laxity in mid-flexion [6–8]. Cross et al. [7] showed an increase of 4.0° at 30° flexion and 1.9° at 60° flexion for 2 mm of joint line elevation (JL), and 6.4° at 30° and 4.0° at 60° flexion for 4 mm. Luyckx et al. [6] found an increase of 64% for 2 mm of JL elevation and 111% for 4 mm. In addition, elevating the JL results in the relaxation of the extension apparatus, which adds to its distension by flexion contracture and exposes it to an active extension deficit, a factor in the recurrence of flexion contracture. Therefore, it is necessary to combine posterior release with additional distal femoral resections to varying degrees on request, depending on the operative findings (extent of residual flexion contracture, the possibility of easily releasing posterior fibers), but the greater the additional resections, the greater the need to be prepared to use a higher prosthetic constraint [39].

There is no consensus on the need for complete correction of flexion contracture intraoperatively. Quite a number of authors have recently reported favorable outcomes for moderate flexion contracture in the early time of a TKA, especially during the first year [48, 49, 58], and even up to 2 [59, 60] or 3 years [47], and even 5 and 10 years [61, 62]. Tanzer and Miller [63], for flexion contracture of less than 30° (12.9° on average), even concluded that trying to correct everything intraoperatively exposes the patient to unjustified additional bone resections. Nevertheless, Mitsuyasu et al. [49] have shown that there is no further improvement between 1 and 2 years and that if there is still flexion contracture of 15° or more at 3 months, the patient will still have flexion contracture; we must therefore strive to achieve no more than 10° flexion contracture at 3 months. On the other hand, other authors insist on the need for complete correction of flexion contracture during surgery [12, 46].

There is a general consensus on postoperative management [12]. As soon as the patient wakes up, it is important to check that there are no neurological deficits (common fibular nerve), as reported in certain series [64], and if there are, to immediately remove any immobilization in full extension and flex the knee. The main threat to evolution is a recurrence of flexion contracture. This recurrence is encouraged by pain, which must be countered. Flexion contracture is in fact the knee’s analgesic position. Immobilization in extension in a posterior plaster cast can be useful for 2–3 days, to avoid immediate recurrence of flexion contracture which is difficult to recover afterward [65–67]. The splint should be removed when the patient is awake and relieved of acute postoperative pain.

When flexion contracture persists after TKA, mobilization under anesthesia may be discussed before 75 days [68]. Indication criteria, technique, and results are not clearly established for flexion contracture. In the mobilization series after TKA, the indication is mainly flexion limitation. Those that also studied extension generally reported improvement in moderate flexion contracture associated with flexion deficit [68–70] but the gain obtained in flexion was the most important [68]. In a review of the literature on mobilization after TKA, Kornuijt et al. [71] concluded that this procedure was effective, especially before 3 months, but the wide variations in indication, timing, and rehabilitation modalities after mobilization meant that no definite conclusions could be drawn.

### Limitations

This systematic review has several limitations. Firstly, flexion measurements are imperfect. Most often, flexion is measured clinically using a 25 cm goniometer, with the greater trochanter, lateral condyle, and lateral malleolus as bony landmarks [72], often in 5° increments [49]. Measurement on a profile radiograph in maximum extension with the heel on support is more accurate [14]. Nevertheless, Lenssen et al. [73] showed significant differences in mobility measurements after TKA, by two experienced observers using a long-leg goniometer (50 cm). With regard to flexion contracture, Jacobs et al. [74] showed that surgeons overestimated flexion contracture before draping and underestimated it once the drapes were in place (flexion of 6.1° ± 6.4°, estimated before draping: 6.9° ± 6.8° and after: 4.3° ± 6.1°). Computer-assisted surgery is a more precise control tool, although less reliable in the sagittal plane than in the coronal plane [75]. Minoda et al. [75] have shown that navigation, which uses different landmarks than conventional techniques, generates 1–4° hyperextension between the femoral and tibial components versus 1° flexion for conventional techniques.

Lastly, the studies in this review focused only on articles that studied TKAs with mechanical alignment. On the other hand, other alignments have been developed, including kinematic alignment (KA), which aims to recreate the patient’s pre-arthritic anatomy, in theory preserving native soft tissue tension and kinematics [76–80]. The correction of flexion contracture in KA TKA has yet to be evaluated and could lead research down a completely different path.

## Conclusion

Our systematic review identified a variety of preoperative and intraoperative steps that influence the incidence and correction of flexion contracture in total knee arthroplasty. The main preoperative factor predicting postoperative flexion contracture is preoperative flexion contracture. Intraoperative steps that have been described to correct flexion contracture are soft tissue release (from the medial and posterior compartments, including osteophyte removal), distal femoral resection, sagittal positioning of the femoral component and posterior condylar resection, which affect the posterior tibial slope. These factors are likely to interact and, therefore, further study is crucial to develop a statistically sound and reliable intraoperative algorithm for surgeons to follow when correcting a fixed flexion deformity. Future studies should measure the range of motion immediately intraoperatively, with standardized and reliable measurement methods such as robotic tools.

## Funding

No funding for this study.

## Conflicts of Interest

Pr Sébastien Lustig is a consultant for Medacta, Heraeus, Corin, Amplitude, Groupe Lépine, Depuy, Smith & Nephew, Stryker Pr Sébastien Lustig receives research support from Corin and Amplitude. Pr Sébastien Lustig is a board member of KSSTA, Maitrise Orthopédique, and JBJS American volume. Dr Sappey-Marinier received personal fees from Medacta and Groupe Lepine. Other authors declare no conflict of interest.

## Data availability statement

Data are available on request from the authors.

## Authors contribution statement

DH and SL compete for the original idea. ESM wrote the systematic review manuscript with the collaboration of AF, JS, CB, and ES. All authors read and approved the final manuscript.

## Ethics approval

The approval of the ethics committee is not required in our institution for this type of study.
